# Correction: A highly efficient heterologous expression platform to facilitate the production of microbial natural products in Streptomyces

**DOI:** 10.1186/s12934-025-02761-6

**Published:** 2025-06-19

**Authors:** Xiuling Wang, Ping Lin, Qiyao Shen, Xueyan Feng, Shouying Xu, Qijun Zhang, Yang Liu, Cailing Ren, Daojing Yong, Qiong Duan, Liujie Huo, Youming Zhang, Gang Li, Jun Fu, Ruijuan Li

**Affiliations:** 1 State Key Laboratory of Microbial Technology, Shandong University, Qingdao, 266237 China; 2 Department of Natural Medicinal Chemistry and Pharmacognosy, School of Pharmacy, Qingdao University, Qingdao, 266071 China

**Microbial Cell Factories (2025) 24:105**

10.1186/s12934-025-02722-z

In this article the wrong figure appeared as Fig. 2; the figure should have appeared as shown below.

Incorrect Fig. 2.

**Fig. 2:**
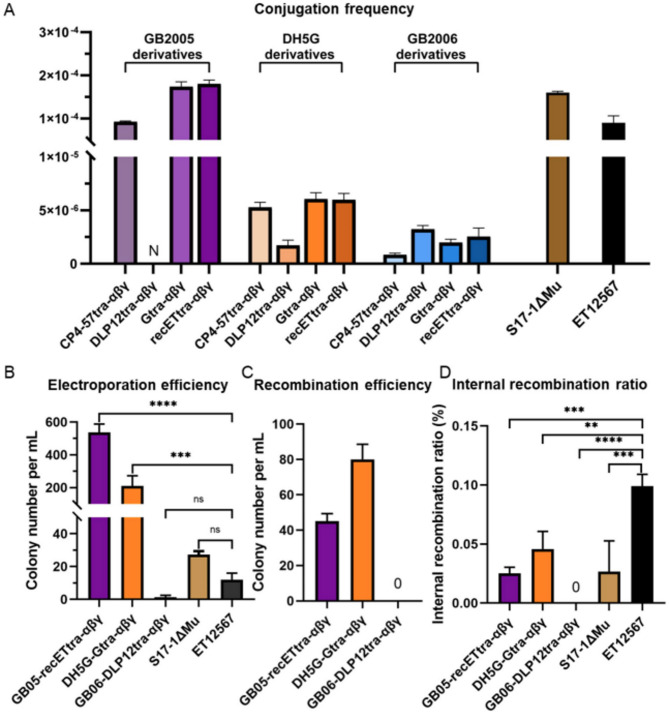
Characterization of the engineered *E. coli* strains. (**A**) Conjugation frequency of plasmid pBAC-sal-phiC31-apra-oriT in transfer from eleven engineered *E. coli* strains to *S. coelicolor* A3(2). Three of the engineered strains were derivatives of *E. coli* GB2005 (purple), with four engineered strains derived each from *E. coli* DH5G (orange) and *E. coli* GB2006 (blue). N represents no data. (**B**) Electroporation efficiency of four engineered strains and *E. coli* ET12567 as determined by the number of colonies grown after transfer of plasmid pBAC-sal-phiC31-apra-oriT (116 kb). (**C**) Recombination efficiency of *E. coli* GB05-recETtra-αβγ, DH5G-Gtra-αβγ, and GB06-DLP12tra-αβγ. The correct recombinant was not obtained in GB06-DLP12tra-αβγ. (**D**) Internal recombination ratio of pBAC-cm-ampF-kan-ampR-repeat in four engineered *E. coli* strains and ET12567. Error bars, SD; *n* = 3; ns, *p* > 0.05; **p < 0.01; ***p < 0.001; ****p < 0.0001

Correct Fig. 2.

**Fig. 2:**
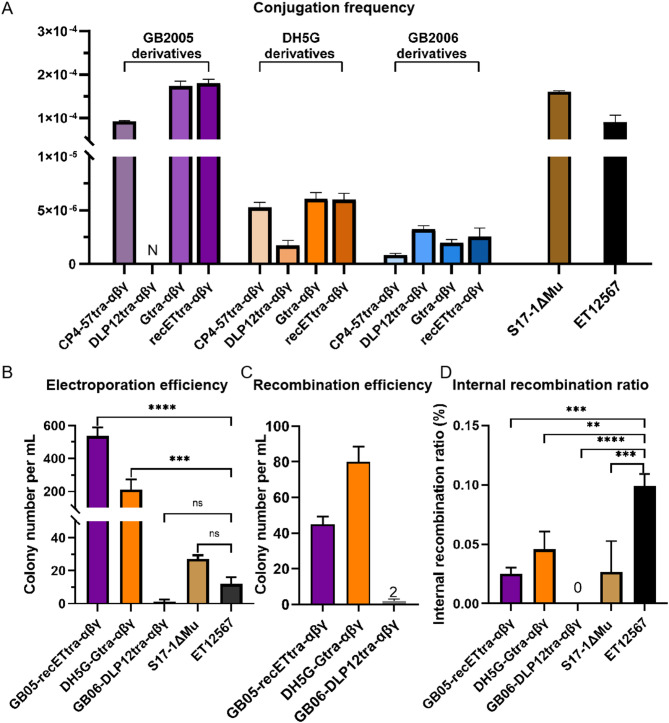
Characterization of the engineered *E. coli* strains. (**A**) Conjugation frequency of plasmid pBAC-sal-phiC31-apra-oriT in transfer from eleven engineered *E. coli* strains to *S. coelicolor* A3(2). Three of the engineered strains were derivatives of *E. coli* GB2005 (purple), with four engineered strains derived each from *E. coli* DH5G (orange) and *E. coli* GB2006 (blue). N represents no data. (**B**) Electroporation efficiency of four engineered strains and *E. coli* ET12567 as determined by the number of colonies grown after transfer of plasmid pBAC-sal-phiC31-apra-oriT (116 kb). (**C**) Recombination efficiency of *E. coli* GB05-recETtra-αβγ, DH5G-Gtra-αβγ, and GB06-DLP12tra-αβγ. The correct recombinant was not obtained in GB06-DLP12tra-αβγ. (**D**) Internal recombination ratio of pBAC-cm-ampF-kan-ampR-repeat in four engineered *E. coli* strains and ET12567. Error bars, SD; *n* = 3; ns, *p* > 0.05; **p < 0.01; ***p < 0.001; ****p < 0.0001

2. The statement in the Result section-“however, recombinants were not obtained with GB06-DLP12tra-αβγ (Fig. 2C)”-should be revised to: “While the recombination efficiency of GB06-DLP12tra-αβγ was inferior to that of DH5G-Gtra-αβγ or GB05-recETtra-αβγ, correct recombinants were indeed generated (Fig. 2C).”

